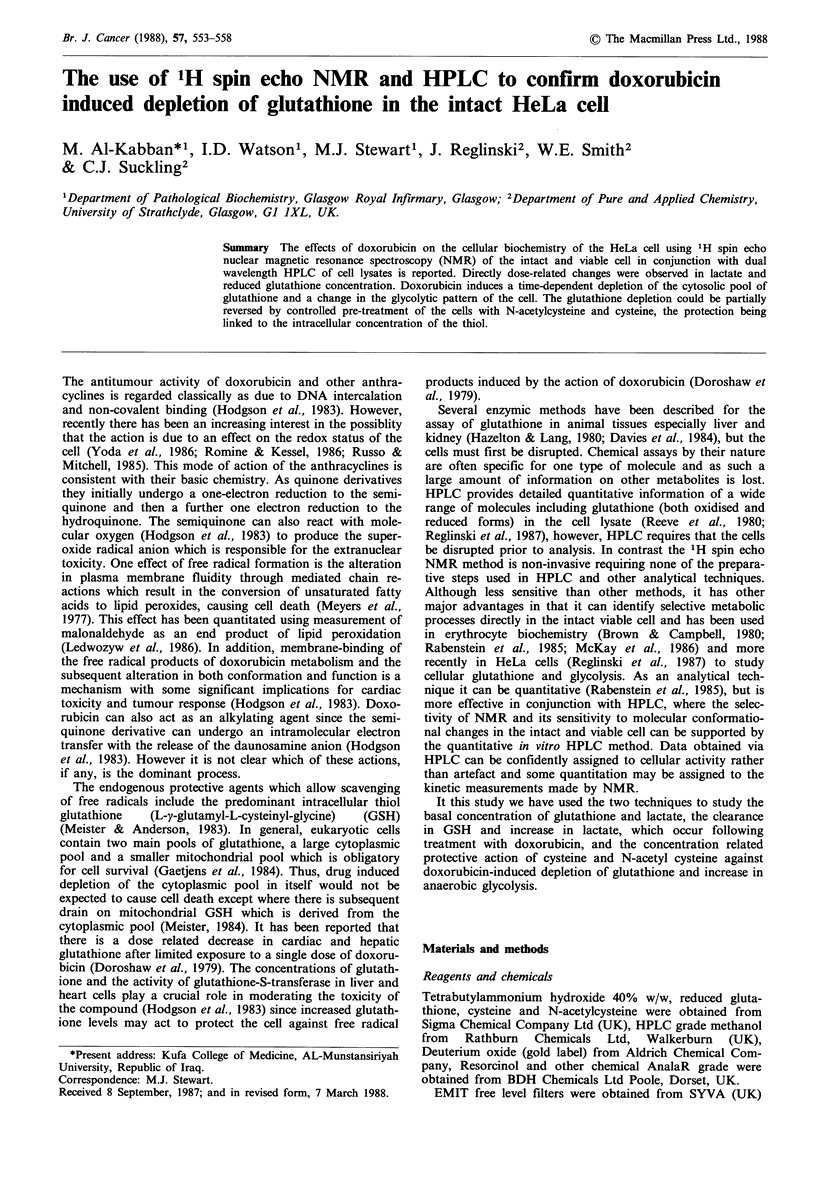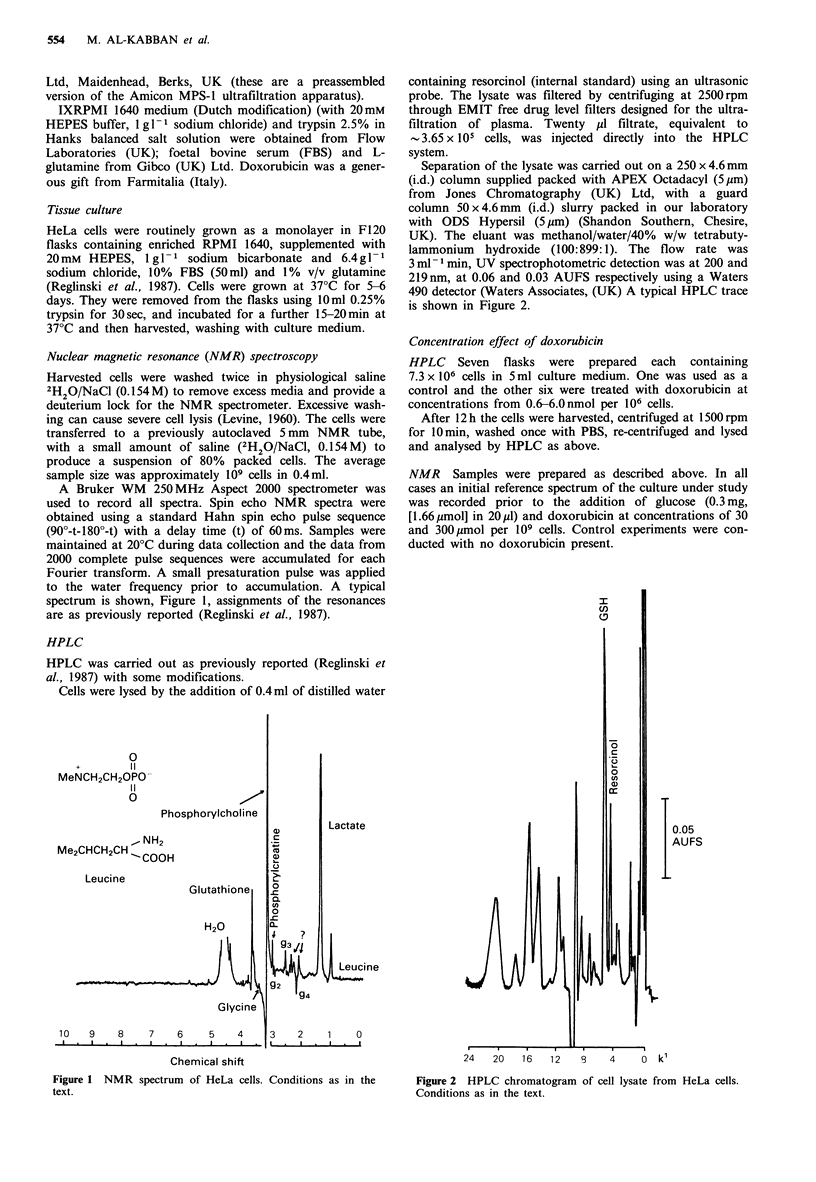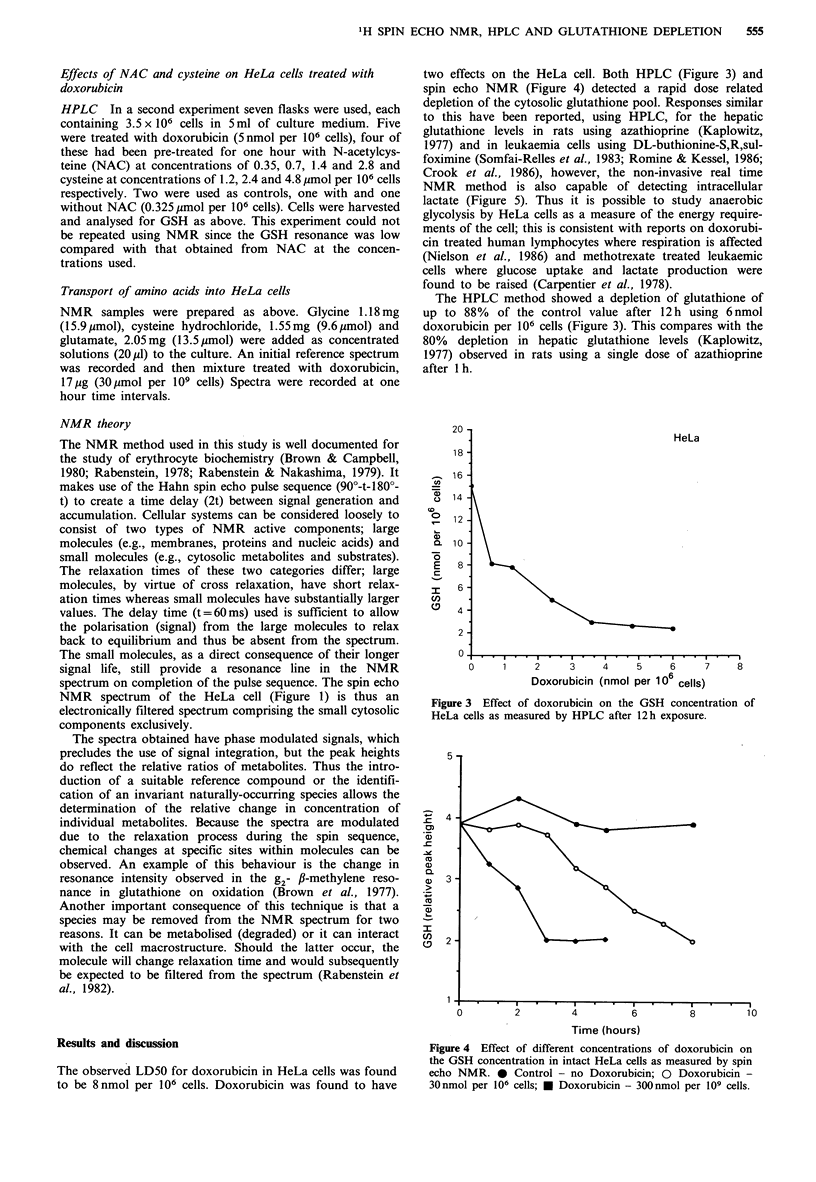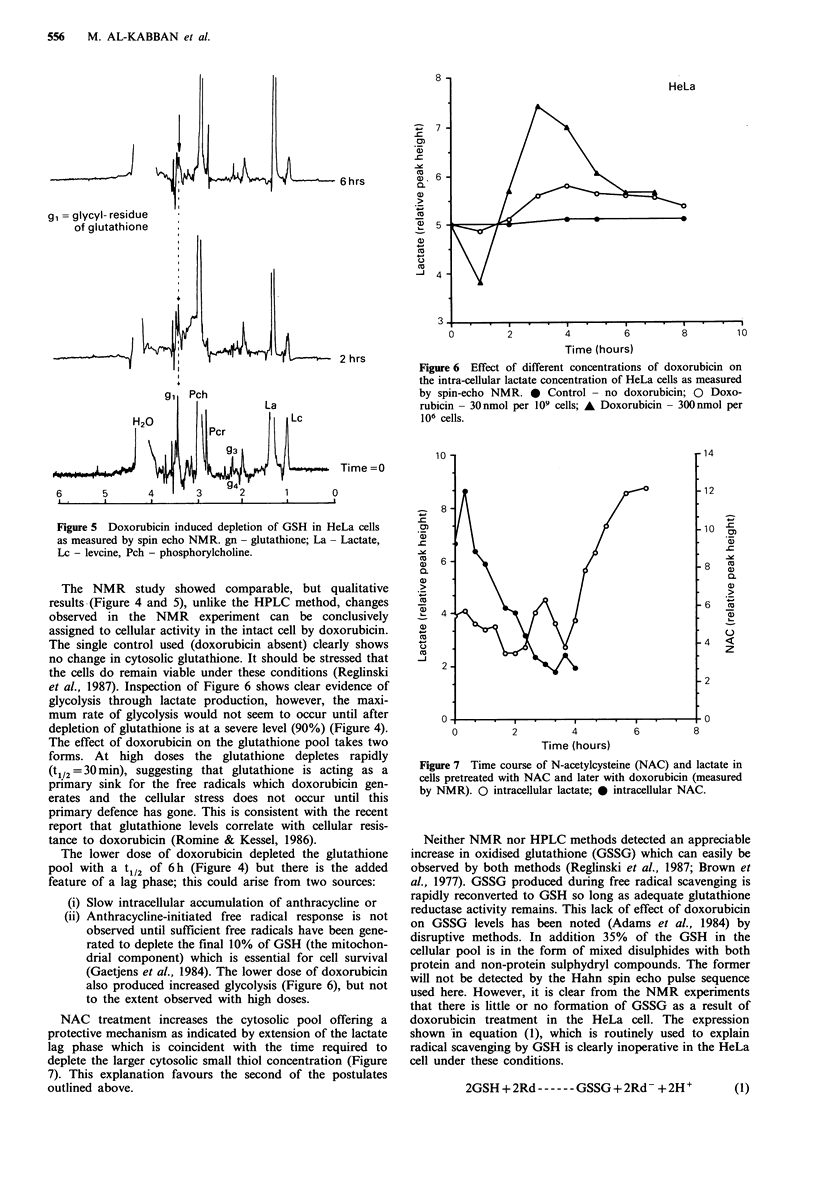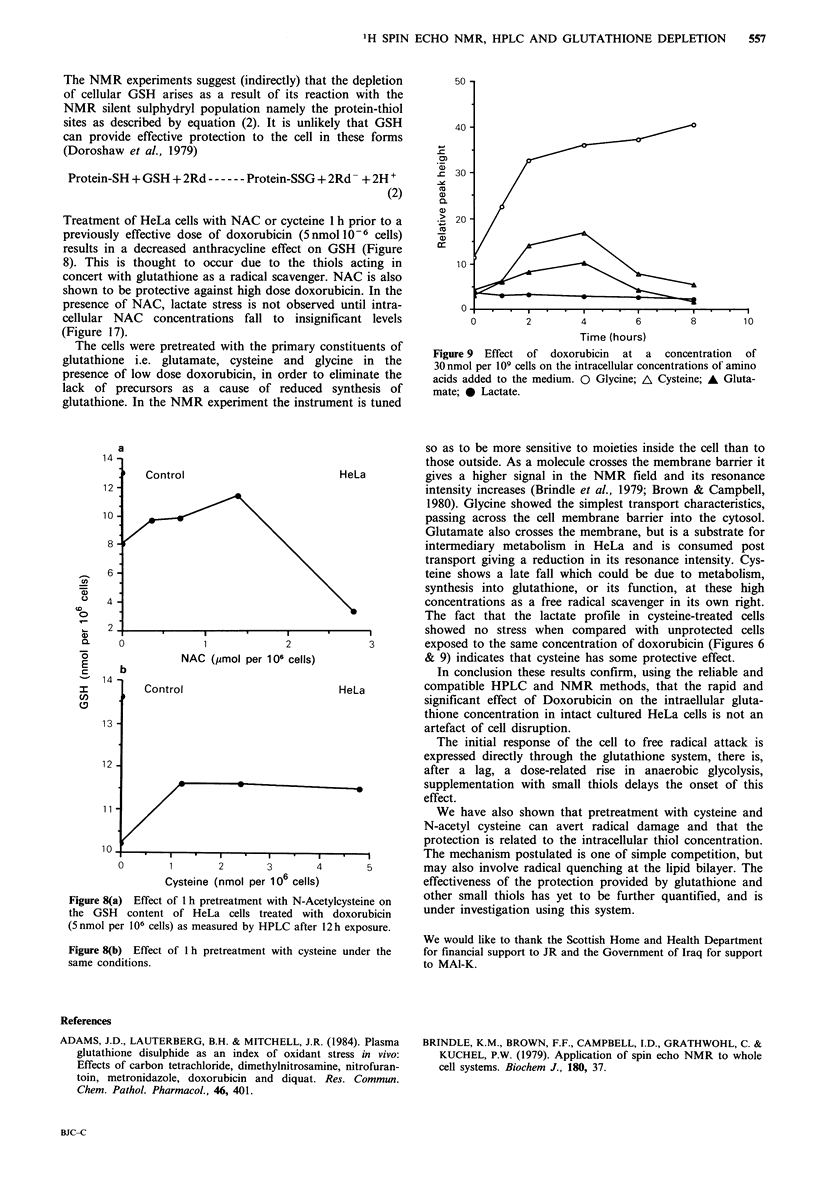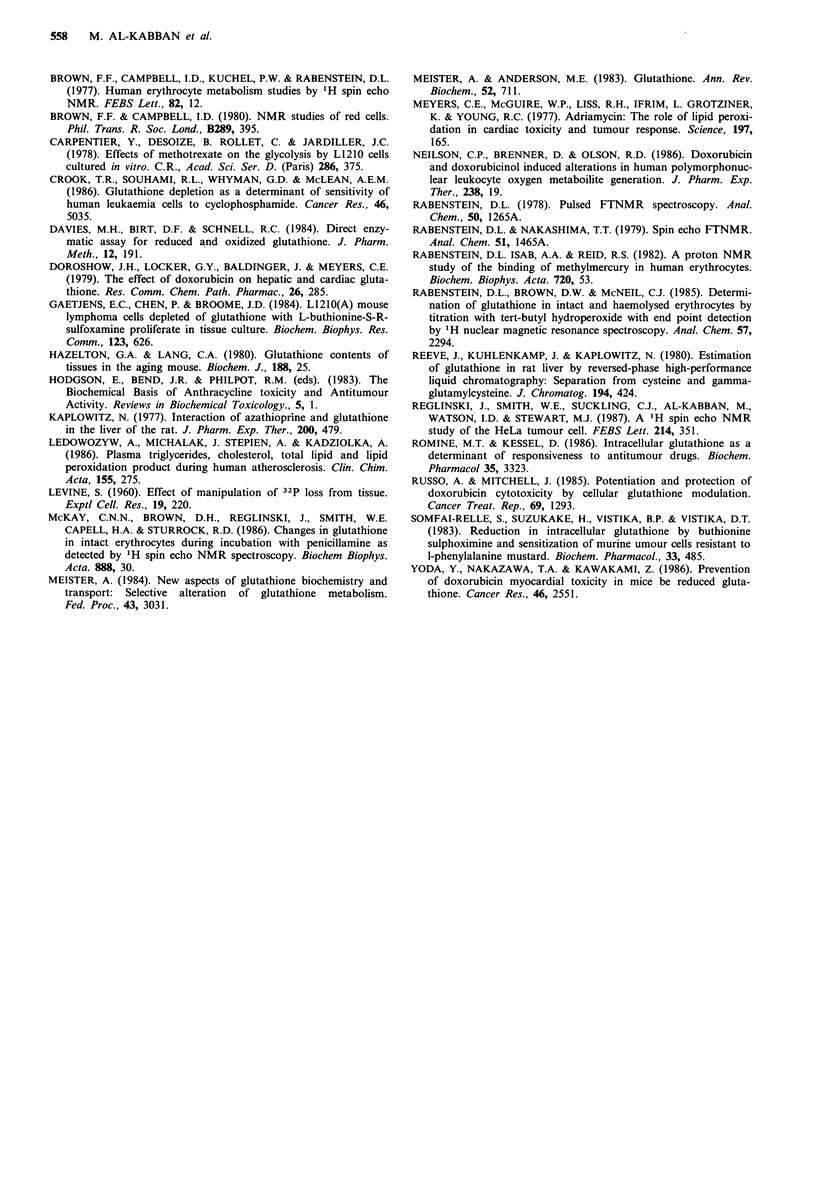# The use of 1H spin echo NMR and HPLC to confirm doxorubicin induced depletion of glutathione in the intact HeLa cell.

**DOI:** 10.1038/bjc.1988.127

**Published:** 1988-06

**Authors:** M. al-Kabban, I. D. Watson, M. J. Stewart, J. Reglinski, W. E. Smith, C. J. Suckling

**Affiliations:** Department of Pathological Biochemistry, Glasgow Royal Infirmary, UK.

## Abstract

The effects of doxorubicin on the cellular biochemistry of the HeLa cell using 1H spin echo nuclear magnetic resonance spectroscopy (NMR) of the intact and viable cell in conjunction with dual wavelength HPLC of cell lysates is reported. Directly dose-related changes were observed in lactate and reduced glutathione concentration. Doxorubicin induces a time-dependent depletion of the cytosolic pool of glutathione and a change in the glycolytic pattern of the cell. The glutathione depletion could be partially reversed by controlled pre-treatment of the cells with N-acetylcysteine and cysteine, the protection being linked to the intracellular concentration of the thiol.


*Br. J. Cancer* (1988), **57**, 553–558 © The Macmillan Press Ltd., 1988

### The use of $^1$H spin echo NMR and HPLC to confirm doxorubicin induced depletion of glutathione in the intact HeLa cell

M. Al-Kabban*[1], I.D. Watson[1], M.J. Stewart[1], J. Reglinski[2], W.E. Smith[2] & C.J. Suckling[2]

[1]*Department of Pathological Biochemistry, Glasgow Royal Infirmary, Glasgow;* [2]*Department of Pure and Applied Chemistry, University of Strathclyde, Glasgow, G1 1XL, UK.*

**Summary** The effects of doxorubicin on the cellular biochemistry of the HeLa cell using $^1$H spin echo nuclear magnetic resonance spectroscopy (NMR) of the intact and viable cell in conjunction with dual wavelength HPLC of cell lysates is reported. Directly dose-related changes were observed in lactate and reduced glutathione concentration. Doxorubicin induces a time-dependent depletion of the cytosolic pool of glutathione and a change in the glycolytic pattern of the cell. The glutathione depletion could be partially reversed by controlled pre-treatment of the cells with N-acetylcysteine and cysteine, the protection being linked to the intracellular concentration of the thiol.

The antitumour activity of doxorubicin and other anthracyclines is regarded classically as due to DNA intercalation and non-covalent binding (Hodgson *et al.*, 1983). However, recently there has been an increasing interest in the possiblity that the action is due to an effect on the redox status of the cell (Yoda *et al.*, 1986; Romine & Kessel, 1986; Russo & Mitchell, 1985). This mode of action of the anthracyclines is consistent with their basic chemistry. As quinone derivatives they initially undergo a one-electron reduction to the semiquinone and then a further one electron reduction to the hydroquinone. The semiquinone can also react with molecular oxygen (Hodgson *et al.*, 1983) to produce the superoxide radical anion which is responsible for the extranuclear toxicity. One effect of free radical formation is the alteration in plasma membrane fluidity through mediated chain reactions which result in the conversion of unsaturated fatty acids to lipid peroxides, causing cell death (Meyers *et al.*, 1977). This effect has been quantitated using measurement of malonaldehyde as an end product of lipid peroxidation (Ledwozyw *et al.*, 1986). In addition, membrane-binding of the free radical products of doxorubicin metabolism and the subsequent alteration in both conformation and function is a mechanism with some significant implications for cardiac toxicity and tumour response (Hodgson *et al.*, 1983). Doxorubicin can also act as an alkylating agent since the semiquinone derivative can undergo an intramolecular electron transfer with the release of the daunosamine anion (Hodgson *et al.*, 1983). However it is not clear which of these actions, if any, is the dominant process.

The endogenous protective agents which allow scavenging of free radicals include the predominant intracellular thiol glutathione (L-γ-glutamyl-L-cysteinyl-glycine) (GSH) (Meister & Anderson, 1983). In general, eukaryotic cells contain two main pools of glutathione, a large cytoplasmic pool and a smaller mitochondrial pool which is obligatory for cell survival (Gaetjens *et al.*, 1984). Thus, drug induced depletion of the cytoplasmic pool in itself would not be expected to cause cell death except where there is subsequent drain on mitochondrial GSH which is derived from the cytoplasmic pool (Meister, 1984). It has been reported that there is a dose related decrease in cardiac and hepatic glutathione after limited exposure to a single dose of doxorubicin (Doroshaw *et al.*, 1979). The concentrations of glutathione and the activity of glutathione-S-transferase in liver and heart cells play a crucial role in moderating the toxicity of the compound (Hodgson *et al.*, 1983) since increased glutathione levels may act to protect the cell against free radical products induced by the action of doxorubicin (Doroshaw *et al.*, 1979).

Several enzymic methods have been described for the assay of glutathione in animal tissues especially liver and kidney (Hazelton & Lang, 1980; Davies *et al.*, 1984), but the cells must first be disrupted. Chemical assays by their nature are often specific for one type of molecule and as such a large amount of information on other metabolites is lost. HPLC provides detailed quantitative information of a wide range of molecules including glutathione (both oxidised and reduced forms) in the cell lysate (Reeve *et al.*, 1980; Reglinski *et al.*, 1987), however, HPLC requires that the cells be disrupted prior to analysis. In contrast the $^1$H spin echo NMR method is non-invasive requiring none of the preparative steps used in HPLC and other analytical techniques. Although less sensitive than other methods, it has other major advantages in that it can identify selective metabolic processes directly in the intact viable cell and has been used in erythrocyte biochemistry (Brown & Campbell, 1980; Rabenstein *et al.*, 1985; McKay *et al.*, 1986) and more recently in HeLa cells (Reglinski *et al.*, 1987) to study cellular glutathione and glycolysis. As an analytical technique it can be quantitative (Rabenstein *et al.*, 1985), but is more effective in conjunction with HPLC, where the selectivity of NMR and its sensitivity to molecular conformational changes in the intact and viable cell can be supported by the quantitative *in vitro* HPLC method. Data obtained via HPLC can be confidently assigned to cellular activity rather than artefact and some quantitation may be assigned to the kinetic measurements made by NMR.

It this study we have used the two techniques to study the basal concentration of glutathione and lactate, the clearance in GSH and increase in lactate, which occur following treatment with doxorubicin, and the concentration related protective action of cysteine and N-acetyl cysteine against doxorubicin-induced depletion of glutathione and increase in anaerobic glycolysis.

### Materials and methods

### Reagents and chemicals

Tetrabutylammonium hydroxide 40% w/w, reduced glutathione, cysteine and N-acetylcysteine were obtained from Sigma Chemical Company Ltd (UK), HPLC grade methanol from Rathburn Chemicals Ltd, Walkerburn (UK), Deuterium oxide (gold label) from Aldrich Chemical Company, Resorcinol and other chemical AnalaR grade were obtained from BDH Chemicals Ltd Poole, Dorset, UK.

EMIT free level filters were obtained from SYVA (UK)

*Present address: Kufa College of Medicine, AL-Munstansiriyah University, Republic of Iraq.
Correspondence: M.J. Stewart.
Received 8 September, 1987; and in revised form, 7 March 1988.

Ltd, Maidenhead, Berks, UK (these are a preassembled version of the Amicon MPS-1 ultrafiltration apparatus).

IXRPMI 1640 medium (Dutch modification) (with 20 mM HEPES buffer, $1\,g\,l^{-1}$ sodium chloride) and trypsin 2.5% in Hanks balanced salt solution were obtained from Flow Laboratories (UK); foetal bovine serum (FBS) and L-glutamine from Gibco (UK) Ltd. Doxorubicin was a generous gift from Farmitalia (Italy).

*Tissue culture*

HeLa cells were routinely grown as a monolayer in F120 flasks containing enriched RPMI 1640, supplemented with 20 mM HEPES, $1\,g\,l^{-1}$ sodium bicarbonate and $6.4\,g\,l^{-1}$ sodium chloride, 10% FBS (50 ml) and 1% v/v glutamine (Reglinski *et al.*, 1987). Cells were grown at 37°C for 5–6 days. They were removed from the flasks using 10 ml 0.25% trypsin for 30 sec, and incubated for a further 15–20 min at 37°C and then harvested, washing with culture medium.

*Nuclear magnetic resonance (NMR) spectroscopy*

Harvested cells were washed twice in physiological saline $^2H_2O$/NaCl (0.154 M) to remove excess media and provide a deuterium lock for the NMR spectrometer. Excessive washing can cause severe cell lysis (Levine, 1960). The cells were transferred to a previously autoclaved 5 mm NMR tube, with a small amount of saline ($^2H_2O$/NaCl, 0.154 M) to produce a suspension of 80% packed cells. The average sample size was approximately $10^9$ cells in 0.4 ml.

A Bruker WM 250 MHz Aspect 2000 spectrometer was used to record all spectra. Spin echo NMR spectra were obtained using a standard Hahn spin echo pulse sequence (90°-t-180°-t) with a delay time (t) of 60 ms. Samples were maintained at 20°C during data collection and the data from 2000 complete pulse sequences were accumulated for each Fourier transform. A small presaturation pulse was applied to the water frequency prior to accumulation. A typical spectrum is shown, Figure 1, assignments of the resonances are as previously reported (Reglinski *et al.*, 1987).

*HPLC*

HPLC was carried out as previously reported (Reglinski *et al.*, 1987) with some modifications.

Cells were lysed by the addition of 0.4 ml of distilled water containing resorcinol (internal standard) using an ultrasonic probe. The lysate was filtered by centrifuging at 2500 rpm through EMIT free drug level filters designed for the ultrafiltration of plasma. Twenty $\mu l$ filtrate, equivalent to $\sim 3.65 \times 10^5$ cells, was injected directly into the HPLC system.

Separation of the lysate was carried out on a 250 × 4.6 mm (i.d.) column supplied packed with APEX Octadacyl (5 $\mu m$) from Jones Chromatography (UK) Ltd, with a guard column 50 × 4.6 mm (i.d.) slurry packed in our laboratory with ODS Hypersil (5 $\mu m$) (Shandon Southern, Chesire, UK). The eluant was methanol/water/40% w/w tetrabutylammonium hydroxide (100:899:1). The flow rate was $3\,ml^{-1}$ min, UV spectrophotometric detection was at 200 and 219 nm, at 0.06 and 0.03 AUFS respectively using a Waters 490 detector (Waters Associates, (UK) A typical HPLC trace is shown in Figure 2.

*Concentration effect of doxorubicin*

*HPLC* Seven flasks were prepared each containing $7.3 \times 10^6$ cells in 5 ml culture medium. One was used as a control and the other six were treated with doxorubicin at concentrations from 0.6–6.0 nmol per $10^6$ cells.

After 12 h the cells were harvested, centrifuged at 1500 rpm for 10 min, washed once with PBS, re-centrifuged and lysed and analysed by HPLC as above.

*NMR* Samples were prepared as described above. In all cases an initial reference spectrum of the culture under study was recorded prior to the addition of glucose (0.3 mg, [1.66 $\mu$mol] in 20 $\mu l$) and doxorubicin at concentrations of 30 and 300 $\mu$mol per $10^9$ cells. Control experiments were conducted with no doxorubicin present.

**Figure 1** NMR spectrum of HeLa cells. Conditions as in the text.

**Figure 2** HPLC chromatogram of cell lysate from HeLa cells. Conditions as in the text.

### *Effects of NAC and cysteine on HeLa cells treated with doxorubicin*

*HPLC* In a second experiment seven flasks were used, each containing $3.5 \times 10^6$ cells in 5 ml of culture medium. Five were treated with doxorubicin (5 nmol per $10^6$ cells), four of these had been pre-treated for one hour with N-acetylcysteine (NAC) at concentrations of 0.35, 0.7, 1.4 and 2.8 and cysteine at concentrations of 1.2, 2.4 and 4.8 $\mu$mol per $10^6$ cells respectively. Two were used as controls, one with and one without NAC (0.325 $\mu$mol per $10^6$ cells). Cells were harvested and analysed for GSH as above. This experiment could not be repeated using NMR since the GSH resonance was low compared with that obtained from NAC at the concentrations used.

### *Transport of amino acids into HeLa cells*

NMR samples were prepared as above. Glycine 1.18 mg (15.9 $\mu$mol), cysteine hydrochloride, 1.55 mg (9.6 $\mu$mol) and glutamate, 2.05 mg (13.5 $\mu$mol) were added as concentrated solutions (20 $\mu$l) to the culture. An initial reference spectrum was recorded and then mixture treated with doxorubicin, 17 $\mu$g (30 $\mu$mol per $10^9$ cells) Spectra were recorded at one hour time intervals.

### *NMR theory*

The NMR method used in this study is well documented for the study of erythrocyte biochemistry (Brown & Campbell, 1980; Rabenstein, 1978; Rabenstein & Nakashima, 1979). It makes use of the Hahn spin echo pulse sequence (90°-t-180°-t) to create a time delay (2t) between signal generation and accumulation. Cellular systems can be considered loosely to consist of two types of NMR active components; large molecules (e.g., membranes, proteins and nucleic acids) and small molecules (e.g., cytosolic metabolites and substrates). The relaxation times of these two categories differ; large molecules, by virtue of cross relaxation, have short relaxation times whereas small molecules have substantially larger values. The delay time (t = 60 ms) used is sufficient to allow the polarisation (signal) from the large molecules to relax back to equilibrium and thus be absent from the spectrum. The small molecules, as a direct consequence of their longer signal life, still provide a resonance line in the NMR spectrum on completion of the pulse sequence. The spin echo NMR spectrum of the HeLa cell (Figure 1) is thus an electronically filtered spectrum comprising the small cytosolic components exclusively.

The spectra obtained have phase modulated signals, which precludes the use of signal integration, but the peak heights do reflect the relative ratios of metabolites. Thus the introduction of a suitable reference compound or the identification of an invariant naturally-occurring species allows the determination of the relative change in concentration of individual metabolites. Because the spectra are modulated due to the relaxation process during the spin sequence, chemical changes at specific sites within molecules can be observed. An example of this behaviour is the change in resonance intensity observed in the $g_2$- $\beta$-methylene resonance in glutathione on oxidation (Brown *et al.*, 1977). Another important consequence of this technique is that a species may be removed from the NMR spectrum for two reasons. It can be metabolised (degraded) or it can interact with the cell macrostructure. Should the latter occur, the molecule will change relaxation time and would subsequently be expected to be filtered from the spectrum (Rabenstein *et al.*, 1982).

### Results and discussion

The observed LD50 for doxorubicin in HeLa cells was found to be 8 nmol per $10^6$ cells. Doxorubicin was found to have two effects on the HeLa cell. Both HPLC (Figure 3) and spin echo NMR (Figure 4) detected a rapid dose related depletion of the cytosolic glutathione pool. Responses similar to this have been reported, using HPLC, for the hepatic glutathione levels in rats using azathioprine (Kaplowitz, 1977) and in leukaemia cells using DL-buthionine-S,R,sulfoximine (Somfai-Relles *et al.*, 1983; Romine & Kessel, 1986; Crook *et al.*, 1986), however, the non-invasive real time NMR method is also capable of detecting intracellular lactate (Figure 5). Thus it is possible to study anaerobic glycolysis by HeLa cells as a measure of the energy requirements of the cell; this is consistent with reports on doxorubicin treated human lymphocytes where respiration is affected (Nielson *et al.*, 1986) and methotrexate treated leukaemic cells where glucose uptake and lactate production were found to be raised (Carpentier *et al.*, 1978).

The HPLC method showed a depletion of glutathione of up to 88% of the control value after 12 h using 6 nmol doxorubicin per $10^6$ cells (Figure 3). This compares with the 80% depletion in hepatic glutathione levels (Kaplowitz, 1977) observed in rats using a single dose of azathioprine after 1 h.

**Figure 3** Effect of doxorubicin on the GSH concentration of HeLa cells as measured by HPLC after 12 h exposure.

**Figure 4** Effect of different concentrations of doxorubicin on the GSH concentration in intact HeLa cells as measured by spin echo NMR. ● Control – no Doxorubicin; ○ Doxorubicin – 30 nmol per $10^6$ cells; ■ Doxorubicin – 300 nmol per $10^9$ cells.

**Figure 5** Doxorubicin induced depletion of GSH in HeLa cells as measured by spin echo NMR. gn – glutathione; La – Lactate, Lc – levcine, Pch – phosphorylcholine.

The NMR study showed comparable, but qualitative results (Figure 4 and 5), unlike the HPLC method, changes observed in the NMR experiment can be conclusively assigned to cellular activity in the intact cell by doxorubicin. The single control used (doxorubicin absent) clearly shows no change in cytosolic glutathione. It should be stressed that the cells do remain viable under these conditions (Reglinski *et al.*, 1987). Inspection of Figure 6 shows clear evidence of glycolysis through lactate production, however, the maximum rate of glycolysis would not seem to occur until after depletion of glutathione is at a severe level (90%) (Figure 4). The effect of doxorubicin on the glutathione pool takes two forms. At high doses the glutathione depletes rapidly ($t_{1/2} = 30$ min), suggesting that glutathione is acting as a primary sink for the free radicals which doxorubicin generates and the cellular stress does not occur until this primary defence has gone. This is consistent with the recent report that glutathione levels correlate with cellular resistance to doxorubicin (Romine & Kessel, 1986).

The lower dose of doxorubicin depleted the glutathione pool with a $t_{1/2}$ of 6 h (Figure 4) but there is the added feature of a lag phase; this could arise from two sources:

(i) Slow intracellular accumulation of anthracycline or
(ii) Anthracycline-initiated free radical response is not observed until sufficient free radicals have been generated to deplete the final 10% of GSH (the mitochondrial component) which is essential for cell survival (Gaetjens *et al.*, 1984). The lower dose of doxorubicin also produced increased glycolysis (Figure 6), but not to the extent observed with high doses.

NAC treatment increases the cytosolic pool offering a protective mechanism as indicated by extension of the lactate lag phase which is coincident with the time required to deplete the larger cytosolic small thiol concentration (Figure 7). This explanation favours the second of the postulates outlined above.

**Figure 6** Effect of different concentrations of doxorubicin on the intra-cellular lactate concentration of HeLa cells as measured by spin-echo NMR. ● Control – no doxorubicin; ○ Doxorubicin – 30 nmol per $10^9$ cells; ▲ Doxorubicin – 300 nmol per $10^6$ cells.

**Figure 7** Time course of N-acetylcysteine (NAC) and lactate in cells pretreated with NAC and later with doxorubicin (measured by NMR). ○ intracellular lactate; ● intracellular NAC.

Neither NMR nor HPLC methods detected an appreciable increase in oxidised glutathione (GSSG) which can easily be observed by both methods (Reglinski *et al.*, 1987; Brown *et al.*, 1977). GSSG produced during free radical scavenging is rapidly reconverted to GSH so long as adequate glutathione reductase activity remains. This lack of effect of doxorubicin on GSSG levels has been noted (Adams *et al.*, 1984) by disruptive methods. In addition 35% of the GSH in the cellular pool is in the form of mixed disulphides with both protein and non-protein sulphydryl compounds. The former will not be detected by the Hahn spin echo pulse sequence used here. However, it is clear from the NMR experiments that there is little or no formation of GSSG as a result of doxorubicin treatment in the HeLa cell. The expression shown in equation (1), which is routinely used to explain radical scavenging by GSH is clearly inoperative in the HeLa cell under these conditions.

$$2GSH + 2Rd \text{------} GSSG + 2Rd^- + 2H^+ \qquad (1)$$

The NMR experiments suggest (indirectly) that the depletion of cellular GSH arises as a result of its reaction with the NMR silent sulphydryl population namely the protein-thiol sites as described by equation (2). It is unlikely that GSH can provide effective protection to the cell in these forms (Doroshaw *et al.*, 1979)

$$\text{Protein-SH} + \text{GSH} + 2\text{Rd} \text{------} \text{Protein-SSG} + 2\text{Rd}^- + 2\text{H}^+ \quad (2)$$

Treatment of HeLa cells with NAC or cycteine 1 h prior to a previously effective dose of doxorubicin (5 nmol $10^{-6}$ cells) results in a decreased anthracycline effect on GSH (Figure 8). This is thought to occur due to the thiols acting in concert with glutathione as a radical scavenger. NAC is also shown to be protective against high dose doxorubicin. In the presence of NAC, lactate stress is not observed until intracellular NAC concentrations fall to insignificant levels (Figure 17).

The cells were pretreated with the primary constituents of glutathione i.e. glutamate, cysteine and glycine in the presence of low dose doxorubicin, in order to eliminate the lack of precursors as a cause of reduced synthesis of glutathione. In the NMR experiment the instrument is tuned so as to be more sensitive to moieties inside the cell than to those outside. As a molecule crosses the membrane barrier it gives a higher signal in the NMR field and its resonance intensity increases (Brindle *et al.*, 1979; Brown & Campbell, 1980). Glycine showed the simplest transport characteristics, passing across the cell membrane barrier into the cytosol. Glutamate also crosses the membrane, but is a substrate for intermediary metabolism in HeLa and is consumed post transport giving a reduction in its resonance intensity. Cysteine shows a late fall which could be due to metabolism, synthesis into glutathione, or its function, at these high concentrations as a free radical scavenger in its own right. The fact that the lactate profile in cysteine-treated cells showed no stress when compared with unprotected cells exposed to the same concentration of doxorubicin (Figures 6 & 9) indicates that cysteine has some protective effect.

**Figure 9** Effect of doxorubicin at a concentration of 30 nmol per $10^9$ cells on the intracellular concentrations of amino acids added to the medium. ○ Glycine; △ Cysteine; ▲ Glutamate; ● Lactate.

**Figure 8(a)** Effect of 1 h pretreatment with N-Acetylcysteine on the GSH content of HeLa cells treated with doxorubicin (5 nmol per $10^6$ cells) as measured by HPLC after 12 h exposure.

**Figure 8(b)** Effect of 1 h pretreatment with cysteine under the same conditions.

In conclusion these results confirm, using the reliable and compatible HPLC and NMR methods, that the rapid and significant effect of Doxorubicin on the intraellular glutathione concentration in intact cultured HeLa cells is not an artefact of cell disruption.

The initial response of the cell to free radical attack is expressed directly through the glutathione system, there is, after a lag, a dose-related rise in anaerobic glycolysis, supplementation with small thiols delays the onset of this effect.

We have also shown that pretreatment with cysteine and N-acetyl cysteine can avert radical damage and that the protection is related to the intracellular thiol concentration. The mechanism postulated is one of simple competition, but may also involve radical quenching at the lipid bilayer. The effectiveness of the protection provided by glutathione and other small thiols has yet to be further quantified, and is under investigation using this system.

We would like to thank the Scottish Home and Health Department for financial support to JR and the Government of Iraq for support to MAl-K.

### References

ADAMS, J.D., LAUTERBERG, B.H. & MITCHELL, J.R. (1984). Plasma glutathione disulphide as an index of oxidant stress *in vivo*: Effects of carbon tetrachloride, dimethylnitrosamine, nitrofurantoin, metronidazole, doxorubicin and diquat. *Res. Commun. Chem. Pathol. Pharmacol.*, **46**, 401.

BRINDLE, K.M., BROWN, F.F., CAMPBELL, I.D., GRATHWOHL, C. & KUCHEL, P.W. (1979). Application of spin echo NMR to whole cell systems. *Biochem J.*, **180**, 37.

BROWN, F.F., CAMPBELL, I.D., KUCHEL, P.W. & RABENSTEIN, D.L. (1977). Human erythrocyte metabolism studies by [1]H spin echo NMR. *FEBS Lett.*, **82**, 12.

BROWN, F.F. & CAMPBELL, I.D. (1980). NMR studies of red cells. *Phil. Trans. R. Soc. Lond.*, **B289**, 395.

CARPENTIER, Y., DESOIZE, B. ROLLET, C. & JARDILLER, J.C. (1978). Effects of methotrexate on the glycolysis by L1210 cells cultured *in vitro*. C.R., *Acad. Sci. Ser. D.* (Paris) **286**, 375.

CROOK, T.R., SOUHAMI, R.L., WHYMAN, G.D. & McLEAN, A.E.M. (1986). Glutathione depletion as a determinant of sensitivity of human leukaemia cells to cyclophosphamide. *Cancer Res.*, **46**, 5035.

DAVIES, M.H., BIRT, D.F. & SCHNELL, R.C. (1984). Direct enzymatic assay for reduced and oxidized glutathione. *J. Pharm. Meth.*, **12**, 191.

DOROSHOW, J.H., LOCKER, G.Y., BALDINGER, J. & MEYERS, C.E. (1979). The effect of doxorubicin on hepatic and cardiac glutathione. *Res. Comm. Chem. Path. Pharmac.*, **26**, 285.

GAETJENS, E.C., CHEN, P. & BROOME, J.D. (1984). L1210(A) mouse lymphoma cells depleted of glutathione with L-buthionine-S-R-sulfoxamine proliferate in tissue culture. *Biochem. Biophys. Res. Comm.*, **123**, 626.

HAZELTON, G.A. & LANG, C.A. (1980). Glutathione contents of tissues in the aging mouse. *Biochem. J.*, **188**, 25.

HODGSON, E., BEND, J.R. & PHILPOT, R.M. (eds). (1983). The Biochemical Basis of Anthracycline toxicity and Antitumour Activity. *Reviews in Biochemical Toxicology.*, **5**, 1.

KAPLOWITZ, N. (1977). Interaction of azathioprine and glutathione in the liver of the rat. *J. Pharm. Exp. Ther.*, **200**, 479.

LEDOWOZYW, A., MICHALAK, J. STEPIEN, A. & KADZIOLKA, A. (1986). Plasma triglycerides, cholesterol, total lipid and lipid peroxidation product during human atherosclerosis. *Clin. Chim. Acta*, **155**, 275.

LEVINE, S. (1960). Effect of manipulation of $^{32}P$ loss from tissue. *Exptl Cell. Res.*, **19**, 220.

McKAY, C.N.N., BROWN, D.H., REGLINSKI, J., SMITH, W.E. CAPELL, H.A. & STURROCK, R.D. (1986). Changes in glutathione in intact erythrocytes during incubation with penicillamine as detected by [1]H spin echo NMR spectroscopy. *Biochem Biophys. Acta.* **888**, 30.

MEISTER, A. (1984). New aspects of glutathione biochemistry and transport: Selective alteration of glutathione metabolism. *Fed. Proc.*, **43**, 3031.

MEISTER, A. & ANDERSON, M.E. (1983). Glutathione. *Ann. Rev. Biochem.*, **52**, 711.

MEYERS, C.E., McGUIRE, W.P., LISS, R.H., IFRIM, L. GROTZINER, K. & YOUNG, R.C. (1977). Adriamycin: The role of lipid peroxidation in cardiac toxicity and tumour response. *Science*, **197**, 165.

NEILSON, C.P., BRENNER, D. & OLSON, R.D. (1986). Doxorubicin and doxorubicinol induced alterations in human polymorphonuclear leukocyte oxygen metaboilite generation. *J. Pharm. Exp. Ther.*, **238**, 19.

RABENSTEIN, D.L. (1978). Pulsed FTNMR spectroscopy. *Anal. Chem.*, **50**, 1265A.

RABENSTEIN, D.L. & NAKASHIMA, T.T. (1979). Spin echo FTNMR. *Anal. Chem.* **51**, 1465A.

RABENSTEIN, D.L. ISAB, A.A. & REID, R.S. (1982). A proton NMR study of the binding of methylmercury in human erythrocytes. *Biochem. Biophys. Acta.* **720**, 53.

RABENSTEIN, D.L., BROWN, D.W. & McNEIL, C.J. (1985). Determination of glutathione in intact and haemolysed erythrocytes by titration with tert-butyl hydroperoxide with end point detection by [1]H nuclear magnetic resonance spectroscopy. *Anal. Chem.* **57**, 2294.

REEVE, J., KUHLENKAMP, J. & KAPLOWITZ, N. (1980). Estimation of glutathione in rat liver by reversed-phase high-performance liquid chromatography: Separation from cysteine and gamma-glutamylcysteine. *J. Chromatog.* **194**, 424.

REGLINSKI, J., SMITH, W.E., SUCKLING, C.J., AL-KABBAN, M., WATSON, I.D. & STEWART, M.J. (1987). A [1]H spin echo NMR study of the HeLa tumour cell. *FEBS Lett.* **214**, 351.

ROMINE, M.T. & KESSEL, D. (1986). Intracellular glutathione as a determinant of responsiveness to antitumour drugs. *Biochem. Pharmacol* **35**, 3323.

RUSSO, A. & MITCHELL, J. (1985). Potentiation and protection of doxorubicin cytotoxicity by cellular glutathione modulation. *Cancer Treat. Rep.*, **69**, 1293.

SOMFAI-RELLE, S., SUZUKAKE, H., VISTIKA, B.P. & VISTIKA, D.T. (1983). Reduction in intracellular glutathione by buthionine sulphoximine and sensitization of murine umour cells resistant to l-phenylalanine mustard. *Biochem. Pharmacol.*, **33**, 485.

YODA, Y., NAKAZAWA, T.A. & KAWAKAMI, Z. (1986). Prevention of doxorubicin myocardial toxicity in mice be reduced glutathione. *Cancer Res.*, **46**, 2551.